# Neglected Dorsal Proximal Interphalangeal Joint Dislocation Treated by Volar Plate Arthroplasty: A Case Series

**DOI:** 10.7759/cureus.60077

**Published:** 2024-05-11

**Authors:** Sunil Gottipati, Deepankar Satapathy, Ranjith K Yalamanchili, Deepak Kumar Maley, Syed Ifthekar, Maheshwar Lakkireddy

**Affiliations:** 1 Orthopaedics, Great Eastern Medical School and Hospital, Srikakulam, IND; 2 Orthopaedics, All India Institute of Medical Sciences, Bibinagar, Bibinagar, IND

**Keywords:** pip fracture dislocation, chronic pip dislocation, extension block pinning for pip joint dislocation, volar plate arthroplasty, neglected pip joint dislocation

## Abstract

Chronic unreduced dislocations of the proximal interphalangeal joint are uncommon, and management principles for these injuries have not been defined. The dislocation can be volar or dorsal and closed reduction is rarely successful owing to soft tissue contractures. Treatment options in literature reviews for such rare injuries included open reduction of pip joint with volar plate arthroplasty, extension block pinning, hemi hamate arthroplasty, pip joint arthrodesis, Suzuki dynamic frame fixation, open reduction and repair of capsule and collateral ligaments with suture anchors. Few cases of amputation following treatment were even reported in literature emphasizing the role of meticulous soft tissue handling in such neglected cases of hand. We report six cases of neglected (more than three months old) dorsal dislocation of the PIP joint of the hand, treated with volar plate arthroplasty and extension block pinning. A functional range of motion with a stable joint can be achieved in such injuries with volar plate arthroplasty, as long as the articular cartilage is relatively preserved and bone loss is <30%.

## Introduction

The proximal interphalangeal (PIP) joint is crucial in daily activities and any impairment in its function will affect the functions of the hand, particularly grip strength [1]. The PIP joint is the most frequent joint in the hand to get injured and dislocation of the PIP joint is not uncommon [2]. Chronic dislocation in the hand is defined as one with the duration of injury over six weeks to presentation [3,4]. The aim of the treatment of any bone and joint fractures of the hand is to achieve an early return to function and pre-injury status. Interphalangeal joint dislocations can be dorsal or volar. Dorsal dislocations are associated with volar plate injury, while volar dislocations are associated with central slip injury. Dorsal dislocation can be pure dislocations and also fracture-dislocations. The treatment algorithm of the same depends on the size of the fracture fragment and the duration of injury to presentation. Fractures involving less than 30% of the volar articular of the middle phalanx are relatively stable and those involving 30-50% require bony fixation or reconstruction of bony architecture for stability [5].

Any chronic presentations (more than six weeks) of hand injury pose a treatment challenge, as they hamper the function of the hand. Stiffness is a common complication with such delayed presentations. In more delayed presentations (more than three months), associated fracture fragments are usually fragile, and in the process of clearing the fibrous tissue they further fragment, leaving fewer options of management viz., either direct volar plate arthroplasty (VPA) or hook of hamate graft reconstruction, basing on stability required due to size of the void created in the base of the middle phalanx. We consider delayed presentations of over more than three months as neglected presentations, and the true picture in such scenarios would be evident only after opening up the joint and clearing the residual fibrous tissue around fracture fragments. In this case series, we present six such cases of neglected PIP dislocations managed.

## Materials and methods

**Figure 1:**
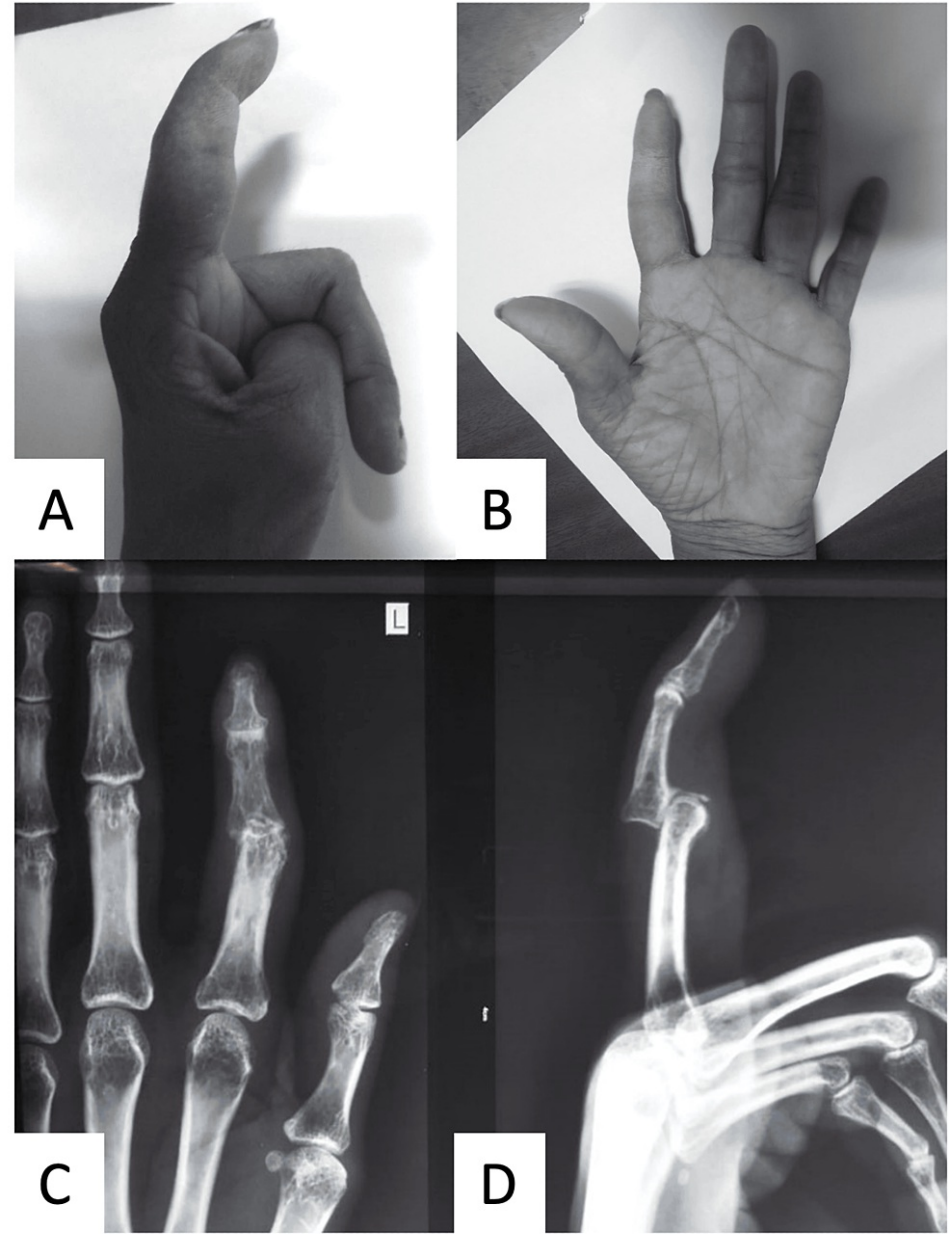
Figures A and B showing the pre-operative clinical image of the index finger with neglected PIP dislocation and Figures C and D showing the radiograph of antero-posterior and lateral projections of the index finger showing dislocation of the PIP joint. PIP: proximal interphalangeal joint

We present six cases of neglected PIP dislocations with a minimum of one-year follow-up after obtaining due consent from the patients. Among the six, four were males and two were females. The average age of the subjects was around 30.16 years (range: 24-38 years), while the average duration of the presentation was 14.6 weeks (range: 13-18 weeks) (Figure 1; Table 1).

**Table 1:** Patient demographics with finger involvement and mechanism of injury IF: index finger; MF: middle finger; RTA: road traffic accident

Case no.	Age in years	Sex	Duration in weeks	Finger involved	Mechanism of Injury	Injury pattern
1	24	Male	16	Left IF	Sports injury	Fracture dislocation with fracture (single chunk attached to volar plate) involvement <30% of base of middle phalanx
2	31	Male	14	Left MF	RTA - Direct injury hand	Pure dislocation
3	28	Female	13	Right IF	Fall	Fracture dislocation with fracture involvement <30% of base of middle phalanx
4	38	Male	18	Right MF	RTA - Direct injury hand	Fracture dislocation with comminuted fracture involvement <30% of base of middle phalanx
5	28	Female	13	Right MF	Fall	Pure dislocation
6	32	Female	14	Left IF	RTA - Direct injury hand	Fracture dislocation with comminuted fracture involvement <30% of base of middle phalanx

**Figure 2:**
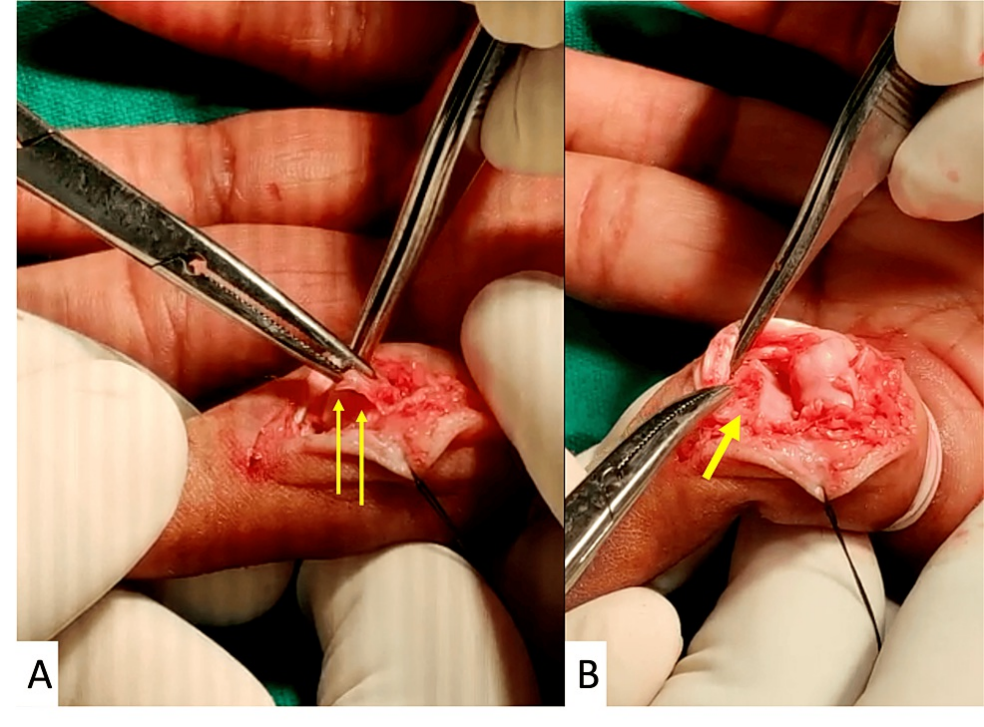
Figure A showing the volar plate interposed in the PIP joint (yellow arrows) after the debridement of fibrous tissue. Figure B showing the bone loss on base of the middle phalanx (yellow arrow) assessed after the debridement of fibrous tissue.

Outcomes were assessed by a range of movements (ROM) at metacarpophalangeal (MCP), PIP joints and Visual Analog Scale (VAS) scores at regular follow-ups of every four weeks until three months, the sixth month and one year thereof. ROM of MCP and PIP joints is measured using iPhone 14 (Apple Inc., Cupertino, California, USA) and Goniometer Plus app (Version 4.0.1). All the cases were done using standard surgical protocol as described here. Elective surgery was planned under wrist block and finger tourniquet. Through the volar approach at the PIP joint, a curvilinear incision was given over the volar aspect of the proximal flexor crease of the index finger with curvature towards the ulnar side of the crease. In cases of fracture comminution, the curve is planned towards the comminuted fragment side to have access to those fragments. Through fine surgical dissection, the underlying tissue is cleared and the volar plate along with the flexor tendon is exposed. The middle phalanx is hyperextended to visualize the joint and cleared of the debris and fibrous tissue in the joint. A volar plate with remnants of fractured fragments was found to interpose in between the joint and it is mobilized carefully to fully expose the joint. In cases of pure dislocations, a volar plate was found interposed in the joint (Figure 2). Lateral accessory ligaments are partially detached to clear the adhesions and mobilize the volar plate. Bone loss in the articular surface of the base of the middle phalanx is visualized and assessed. The dorsal dislocation is reduced by hyperextension at PIP but was found unstable in all the cases due to lack of volar plate support. The volar plate was mobilized to create a loose flap-like structure. The remaining base of the middle phalanx is freshened and to create a transverse groove for the reattachment of the volar plate (Figure 2).

**Figure 3:**
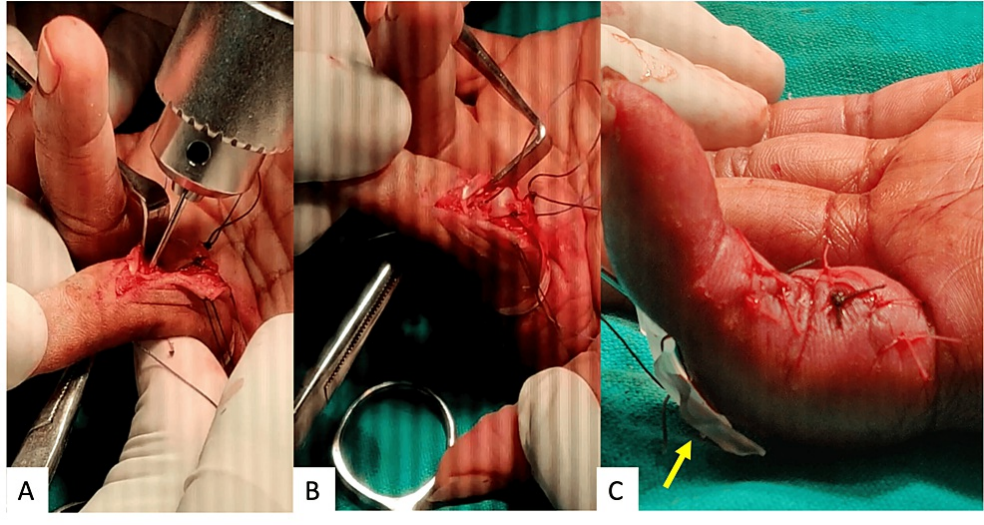
Figure A showing drill holes made through the freshened edges in the area of bone loss as close to the articular cartilage as possible. Figure B showing sutures from the volar plate being passed through the bony tunnel. Figure C showing tightened pull-out sutures over dorsal aspect of the finger over a rubber drain (yellow arrow) and surgical wound closure.

The groove must be perpendicular to the long axis of the middle phalanx in order to avoid lateral angulation. Keeping the phalanx hyperextended, a 1mm k wire is drilled through the base of the fractured phalanx. The drill holes are positioned as close to the articular cartilage as possible in order to prevent later subluxation (Figure 3). If the drill holes are not immediately adjacent to the articular cartilage, the pull of the flexor digitorum superficialis (FDS) will cause dorsal subluxation.

The volar plate is sutured end on with 3-0 Vicryl (Polyglycolic acid, Ethicon Inc., New Jersey, USA) to make two ends from the distal portion of the volar plate. A 16 g hollow needle is placed in the drilled proximal fractured area of the middle phalanx and the ends of the Vicryl are brought through the dorsum of the finger at the middle phalanx. Traction on the four suture threads will draw the volar plate securely into the groove. The volar plate is seated in the groove and the reduced joint is confirmed with image intensification. Confirm that the volar plate has entered the prepared groove. Sutures are tightened over the dorsum of the middle phalanx over a suture button or a rubber drain (Figure 3).

The PIP joint is positioned in 30 degrees of flexion and an extension block k wire is passed through the middle phalanx to the proximal phalanx after confirming through an image intensifier. The volar skin is carefully sutured with 3-0 silk.

An extension block splint was applied and the sutures were removed at two weeks post-operatively. The k wire is removed after four weeks postoperatively. Active physiotherapy of the PIP joint was encouraged.

## Results

**Figure 4:**
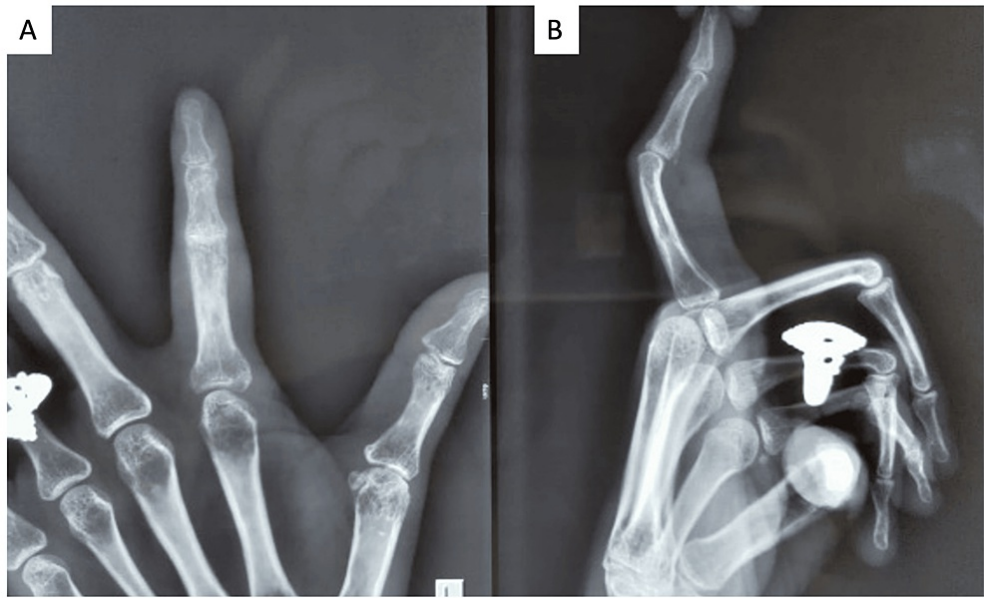
Figure A shows a one-year follow-up radiograph antero-posterior view of the hand focused on the index finger. Figure B shows the lateral view of the index finger showing no subluxation or dislocation.

The average follow-up period of the cases included was 13.4 months (12.3-15.6 months). Radiographs assessed at regular intervals showed no subluxation or dislocation among the study patients (Figure 4). The average VAS score pre-operatively was 8.7±0.4, at four weeks was 8.2±0.6, at eight weeks was 5.8±1.6, at 12 weeks was 4.6±2.4 and it improved to 2.1±0.3 at one year of follow-up. All six patients had mild swelling around the incision region compared to the uninjured site even at the final follow-up. Patients did not have any complaints of swelling like pain or tenderness due to swelling that bothered their daily activities. ROM at MCP and PIP joints of the subjects are tabulated (Table 2).

**Table 2:** Clinical ROM at MCP and PIP joints pre-operatively and at a one-year follow-up ROM: range of movement; MCP: metacarpophalangeal joint; PIP: proximal interphalangeal joint Note: All values of the ROM are measured in degrees.

	MCP Joint ROM		PIP joint ROM		
Case no.	Pre-operative	One year follow-up	Pre-operative	One year follow-up	Extension lag at final follow-up
1	46	71	11	79	5
2	51	76	6	81	7
3	44	73	13	72	9
4	61	79	8	66	11
5	56	74	14	84	3
6	52	72	12	72	7

**Figure 5:**
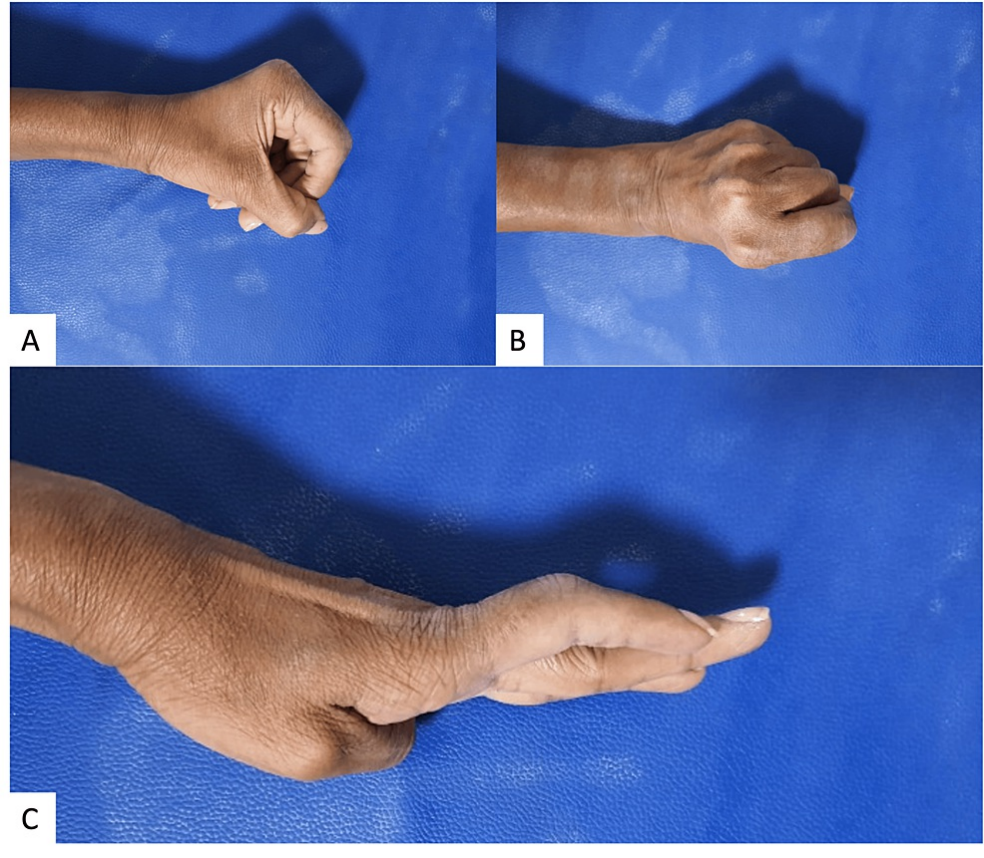
Figures A and B showing the grip of the hand with flexion of fingers. Figure C showing the mild flexion contracture at the PIP joint of the index finger (11 degrees) at a one-year follow-up. PIP: proximal interphalangeal joint

The average pre-operative ROM at the MCP joint was 51.6 degrees and it improved to 74.16 degrees at one year follow-up. The average ROM at the PIP joint was 10.66 degrees pre-operatively which improved to 75.6 degrees at the final follow-up. The average flexion contracture at the PIP joint was 7 degrees. Flexion contracture improved drastically when compared to the immediate postoperative period. Average flexion contracture at four weeks was 21.8 degrees ± 6.9 and at eight weeks was 14.3 degrees ± 4.2. At 12 weeks follow-up, it improved to an average of 9.3 degrees ± 3.4 to 7 degrees ± 4 at one-year follow-up (Figure 5).

Active physiotherapy and wax bath therapy helped patients regain the ROM at the finger. None of the cases in the series had any wound necrosis or wound infections. K wire tract used for the extension block splint had no signs of pin tract infection as well. Two cases had hypoaesthesia on surgical scar even at one-year follow-up.

## Discussion

Articular fractures of the hand need to be attended to at the earliest to achieve the goal of early mobilization for the best possible outcome. Assessment of articular fractures of fingers of the hand with respect to size of fracture fragments, associated wound if any, stability of the joint, and underlying capsular or volar plate damage is very much important while planning the management. Treatment options range from ligamentotaxis with mini external fixators [6] or Joshi External Stabilizing System (JESS), Suzuki frame [7] assembly to simple k-wire fixations [8], trans-osseous fixation [9] and 1.5 to 2.0 mm fragment fixation systems [6]. If there is no associated dislocation of the PIP joint, it can be managed with closed methods and preferably using the principle of ligamentotaxis [10]. If there is an associated dislocation or dislocation of the PIP joint only, it needs to be addressed with respect to the stability of the joint. Conservative management of these injuries and traction with k wire reported union but not stability and functional ROM [11]. Open reduction is recommended and fractured fragments can be fixed with 1.5 or 2.0 mm screws or a min fragment buttress plate over a volar plate area to reduce comminuted fragments [12]. When the fractured fragments with the attached volar plate are not amenable for osteosynthesis, reconstruction of the volar plate by VPA [3] or hook of the hamate graft [11,13,14] reconstruction of the base of middle phalanx is the standard of care. Tenodesis with FDS and arthrodesis of PIP joint were reported by few authors in the literature [15]. Evidence for successful outcome of VPA in bone loss of less than 30% has been reported so far [16]. The technique of VPA was described in 1980, where comminuted fragments were all excised and the volar plate was repaired to a bony trough on the middle phalanx [17]. They have reported dorsal dislocation and subluxations that were proportional to the size of comminution. In our series, we have performed VPA in all six reported cases and observed no cases of instability. Understanding of counter force of FDS and bony architecture required for inherent stability of PIP joint, treatment algorithm for fracture-dislocations was recommended based on the size of the base of middle phalanx fracture. In our cases, comminuted fragments were completely excised, and the volar plate was repaired. In two cases where a fragment was attached to the volar plate, pull-through sutures were used from the volar plate through the trough of the middle phalanx to manage the fracture reduction and achieve stability. Although VPA yields a good outcome, care to repair the volar plate through the bone trough in cases of a fractured dislocation or as near to the articular cartilage in cases of pure dislocation is mandatory. More distal attachment of the volar plate will result in imbalanced volar pull. However, flexion contracture seen in all the cases in our series could be due to neglected presentation which has preoperative scarring of tissues and added post-operative scarring along with articular changes in the PIP joint due to chronicity and not due to malpositioning of the volar plate. Flexion contracture as a result of VPA was commonly reported in the literature, particularly in chronic presentations. Wollstein et al. have reported that reoperation for contracture release was required in four patients who had residual flexion contracture among 54 patients of their series operated by VPA for chronic PIP dislocation [18]. Lee et al. have performed the repairs using Mitek suture anchors and also have observed flexion contracture, ruling out that the technique of repair has no role in avoiding flexion contractures [19]. Kaneshiro et al. have reported an average of 9 (range 0-20) degrees of flexion contracture after VPA for chronic PIP dorsal dislocations in their series [20]. Our series also has a similar range of flexion contractures as reported in the literature. Melone described that a minor residual flexion contracture as a surgically created check rein to dorsal instability and not the complication of VPA [21].

It is emphasized that the volar plate is the key restraint to dorsal forces [15]. Frueh et al. opined that due to the chronicity of injury, the volar plate retracts and thins out, decreasing its healing potential and thereby affecting its stabilizing ability [22]. An analysis of the volar plate by imaging (MRI or ultrasonography) in the pre-operative and post-operative periods would have assessed the status of the volar plate critically. Results in our case series with no evidence of subluxation have shown the stabilizing ability of the volar plate repaired in the chronic presentations as well. It is very difficult to comment on the thinning of the volar plate, as it is very subjective and there are no objective parameters for evaluation of its structure.

Hook of hamate or hemi hamate graft (HHG) used in the reconstruction of the base of the middle phalanx wherein >30% bone loss was observed have reported complications ranging from stiffness to graft resorption as well [23]. Williams et al. recommended HHG, particularly in cases where >50% of bone loss is observed [5]. Technical steps with the harvest of graft and positioning the graft correctly to mimic articular congruity are the key challenges associated with HHG [16]. VPA relatively has a lesser learning curve and is easy to reproduce procedure with fair to good outcomes and more studies are required to analyze its role in cases with 30-50% of bone loss.

The limitations of this study are a smaller sample size, retrospective study, and lack of proper mechanism to explain residual flexion contracture.

## Conclusions

Neglected dorsal PIP joint dislocations present a treatment challenge, particularly when presenting beyond three months post-injury. VPA combined with extension block pinning emerged as a viable treatment option in our case series. Despite the complexity of the injuries, VPA facilitated stable joint reconstruction, resulting in functional ROM and improved outcomes. Our findings underscore the significance of meticulous surgical technique and early intervention in addressing neglected PIP dislocations. Further research is warranted to validate the efficacy of VPA in cases with varying degrees of bone loss and to refine treatment algorithms for these challenging injuries.
